# Phenotypic Presentation and Longitudinal Characterization of Hereditary ATTRv Amyloidosis in Previously Undiagnosed Family Members

**DOI:** 10.1016/j.jacadv.2025.102036

**Published:** 2025-07-24

**Authors:** Luca Fazzini, Matteo Castrichini, Yan Li, Jose De Melo, Marta Figueiral, Jenny J. Cao, Eric W. Klee, Christian Cadeddu Dessalvi, Martha Grogan, Angela Dispenzieri, Naveen L. Pereira

**Affiliations:** a Department of Cardiovascular Medicine, Mayo Clinic, Rochester, Minnesota, USA; b Department of Medical Sciences and Public Health, University of Cagliari, Cagliari, Italy; c Department of Quantitative Health Science, Division of Clinical Trials and Biostatistics, Mayo Clinic, Rochester, Minnesota, USA; d Department of Quantitative Health Science, Division of Computational Biology, Mayo Clinic, Rochester, Minnesota, USA; e Division of Hematology, Mayo Clinic, Rochester, Minnesota, USA

**Keywords:** amyloidosis, asymptomatic amyloidosis, cardiac amyloidosis, family members, gene carriers, transthyretin

## Abstract

**Background:**

Clinical characteristics, cardiac disease progression, and outcomes of “previously undiagnosed” family members of patients with hereditary transthyretin amyloid cardiomyopathy (ATTRv-CM) with pathogenic or likely pathogenic transthyretin (TTR) variants (genotype positive or G+) are unknown despite prognostic and therapeutic implications.

**Objectives:**

The objectives of this study are to describe the phenotypic presentation and report longitudinal assessment, including cardiac imaging of ATTRv G+ family members.

**Methods:**

Demographic, electrocardiographic, genetic, and imaging (echocardiography, cardiac technetium-99m pyrophosphate, and magnetic resonance imaging) data were abstracted and analyzed from the electronic health records.

**Results:**

There were 85 G+ family members, with the most common genotypes being Val50Met (29.4%) and Thr60Ala (28.2%). The mean age was 48.5 ± 11.7 years, 38.8% were male, and 17.9% and 15.5% had a diagnosis of peripheral neuropathy and carpal tunnel syndrome, respectively. The median follow-up was 6.8 years (Q1-Q3: 4.1-9.7), over which 55 patients had follow-up imaging studies. Left ventricular ejection fraction reduction (63 ± 4 to 61 ± 4, *P* = 0.014) and progressive septal wall thickening (9.4 ± 1.6 to 10.2 ± 2.4, *P* = 0.037) were observed. There were only 6 (10.9%) patients who developed at least 2 abnormal echocardiographic changes consistent with cardiac disease progression. The risk of developing peripheral neuropathy during follow-up was 25.5% (95% CI: 8.9%-42.1%; *P* = 0.004), but none were diagnosed with heart failure.

**Conclusions:**

Previously undiagnosed ATTRv G+ family members have a greater prevalence and incidence of symptomatic neurological rather than cardiac disease, and the progression of cardiac disease was limited, which has implications for treating these patients preemptively.

Cardiac amyloidosis (CA) is a well-known cause of heart failure and a life-threatening disease.^1^, ^2^, ^3^ Early diagnosis of transthyretin amyloidosis (ATTR), which is a common cause of CA, has become especially relevant given recent advances in diagnosis and therapy.^4^ Cardiomyopathy (CM) due to ATTR is classified as either wild-type or hereditary (ATTRv-CM).^5^ ATTRv is an autosomal-dominant disease and over 130 transthyretin (*TTR*) variants have been identified that have ethnic and geographic distribution.^6^, ^7^, ^8^ Different TTR genotypes are associated with varying phenotypes.^9^, ^10^, ^11^ The correlation between genetic heterogeneity, variability in disease onset, and expression remains an active area of investigation.^12^

Genetic testing is recommended in patients diagnosed with ATTR-CM to distinguish between wild-type and hereditary disease.^4^^,^^13^, ^14^, ^15^ Identifying a pathogenic (P) or likely-pathogenic (LP) variant not only enables a diagnosis of ATTRv-CM but also allows for the cascade testing of first-degree relatives.^16^ However, managing genotype-positive (G+) (carriers of a P or LP variant) remains a challenge due to a lack of information on the natural course of the disease and the uncertain cardiac penetrance of these *TTR* variants. Characterizing the phenotype, understanding outcomes, and identifying high-risk individuals will inform strategies for not only monitoring but also potentially treating *TTR* G+ carriers. The purpose of this study was to characterize the phenotypic presentation and analyze the longitudinal, clinical, and cardiac imaging assessment in previously undiagnosed *TTR* G+ individuals evaluating their natural history.

## Methods

### Study population

The Institutional Review Board of the Mayo Clinic Foundation provided appropriate ethical oversight and approved this observational study. Among the overall population affected by amyloidosis followed at the Mayo Clinic, Rochester, previously undiagnosed family members of probands who were diagnosed with ATTRv and underwent genetic testing were identified from January 1998 to October 2023. Family members who had never undergone any prior clinical evaluation or genetic screening for ATTR and without evidence of ATTR-CM at baseline screening (G+) were included in the study and were defined as “previously undiagnosed.” The absence of such a previous diagnosis was confirmed by performing a systematic review of medical records. Pertinent demographic, clinical, electrocardiographic (ECG), and imaging data available at our institution closest to the date of genetic testing for these patients were extracted from the electronic medical record. Patients without cardiac imaging at baseline were excluded since the baseline cardiac phenotype could not be determined.

### Genetic testing

Genomic DNA obtained from submitted samples was enriched for targeted regions using a hybridization-based protocol. Sequence analysis and deletion/duplication testing of the *TTR* gene were performed. A bidirectional sequence analysis was performed to test for the presence of variants in all coding regions and intron/exon boundaries of the *TTR* gene. A P/LP variant in the *TTR* gene was defined as per the American College of Medical Genetics and Genomics standard criteria.^17^

### Artificial intelligence–enabled electrocardiography

Given its potential predictive role and easy routine applicability at our institution, a previously developed and validated artificial intelligence (AI) algorithm to diagnose CA was applied to the baseline ECG of the G+ carriers.^18^^,^^19^ An AI-ECG was considered positive when the probability threshold to detect patients with CA by the Youden index was 48.5% or higher.

### Statistical analysis

Descriptive statistics were used to summarize the characteristics of the study population. Continuous variables are reported as mean (SD) or median with 25th-75th percentiles (Q1-Q3), and categorical variables are reported as count (percentage). To evaluate if clinical and echocardiographic characteristics are changed between baseline and follow-up, continuous variables were tested using paired two-sided t-tests, whereas binary variables were tested if their proportional difference between baseline and follow-up was different from 0 using a Z-test. The changes from baseline results are presented as the mean or proportional difference between baseline and follow-up with a 95% CI. For all tested hypotheses, the significance level of 0.05 was considered, and multiple testing was adjusted by the Bonferroni correction method. All analyses were performed using R (version 4.2.2, R Core Team [2022]. R: A language and environment for statistical computing).

## Results

Among the overall ATTR population reviewed (n = 1802; 344 were affected by ATTRv), 85 ATTRv carriers were identified with P/LP *TTR* variants and cardiac imaging assessment at the time of genetic testing (Supplemental Figure 1). Among these, 80 patients had echocardiography, 34 patients had pyrophosphate (PYP) scintigraphy, and 29 patients had both echocardiography and PYP scintigraphy at baseline. Baseline cardiac assessment was performed around the time of the genetic testing report, with a median time of 1 day (Q1-Q3: 0-77).

### Genotypic and phenotypic characteristics of ATTR G+ individuals

Baseline characteristics of the patient population are reported in Table 1. The mean age was 48.5 ± 11.7 years, 38.8% were males, and 90.6% were White. Comorbidities included spinal stenosis (2.4%), chronic kidney disease (1.2%), peripheral neuropathy (17.9%), and bilateral carpal tunnel syndrome (15.5%). One patient had undergone permanent pacemaker implantation and 2.4% had a prior history of syncope. Troponin T levels were elevated in 17.4% of patients (the upper limit of normal was: 10 ng/L for women and 15 ng/L for men), and median N-terminal pro–B-type natriuretic peptide in the cohort was normal at 51 ng/L (Q1-Q3 25-88).

**Table 1:** Baseline Characteristics of the Overall G + ATTRv Cohort of Family Members, as Well as the Populations With or Without Follow-Up Clinical and Imaging Studies

	Overall (N = 85)	No Follow-Up (n = 30)	With Follow-Up (n = 55)	*P* Value^a^
Age (y)	48.5 (11.7)	49.6 (14.1)	48.0 (10.3)	0.59
Male, n (%)	33 (38.8)	8 (26.7)	25 (45.5)	0.089
White, n (%)	77 (90.6)	26 (86.7)	51 (92.7)	0.44
Proband’s cardiac involvement, n (%)	47 (55.3)	14 (46.7)	33 (60.0)	0.24
Proband’s neurological involvement, n (%)	51 (60.0)	21 (70.0)	30 (54.5)	0.16
Proband’s organ involvement unknown, n (%)	10 (11.8)	4 (13.3)	6 (10.9)	0.74
First-degree relatives, n (%)	59 (69.4)	22 (73.3)	37 (67.3)	0.56
Second-degree relatives, n (%)	26 (30.6)	8 (26.7)	18 (32.7)	-
Blood test				
WBC (x10^3^/μL)^b^	6.1 (5.1-6.9)	5.9 (4.7-6.8)	6.1 (5.1-7.1)	0.096
Hb (g/dL)	13.9 (1.4)	13.8 (1.3)	14.0 (1.4)	0.67
PLT (x10^3^/μL)^b^	231.0 (208.0-251.5)	226.0 (195.0-243.8)	236.5 (210.2-252.5)	0.18
Positive troponin T, n (%)	12 (17.4)	7 (31.8)	5 (10.6)	0.043
NT-proBNP (ng/L)^b^	51.0 (25.0-88.0)	52.0 (32.5-96.5)	48.0 (25.0-85.5)	0.57
Comorbidities				
Spinal stenosis, n (%)	2 (2.4)	0 (0.0)	2 (3.6)	0.54
Peripheral neuropathy, n (%)	15 (17.9)	5 (17.2)	10 (18.2)	1.00
CKD n (%)	1 (1.2)	0 (0.0)	1 (1.8)	1.00
Bilateral carpal tunnel syndrome, n (%)	13 (15.5)	4 (13.8)	9 (16.4)	1.00
Pacemaker, n (%)	1 (1.2)	1 (3.4)	0 (0.0)	0.35
Syncope, n (%)	2 (2.4)	0 (0.0)	2 (3.6)	0.54
ECG				
Atrial fibrillation, n (%)	3 (3.6)	0 (0.0)	3 (5.5)	0.55
AVB, n (%)	3 (4.4)	1 (5.0)	2 (4.2)	1.00
Low voltage, n (%)	1 (1.5)	0 (0.0)	1 (2.1)	1.00
Pseudoinfarct pattern, n (%)	3 (4.4)	0 (0.0)	3 (6.2)	0.55
AI amyloid probability above threshold, n (%)	5 (5.9)	2 (6.7)	3 (5.5)	1.00
Imaging				
Echocardiography, n (%)	80 (94.1)	27 (90.0)	53 (96.4)	0.34
PYP scintigraphy, n (%)	34 (40.0)	14 (46.7)	20 (36.4)	0.35
Cardiac magnetic resonance, n (%)	4 (4.7)	0 (0.0)	4 (7.3)	0.29
Baseline echocardiographic features				
LVEF	62.6 (7.4)	61.0 (11.6)	63.3 (4.3)	0.36
Posterior wall thickness (mm)	9.1 (1.4)	8.8 (1.1)	9.3 (1.5)	0.18
Septal wall thickness (mm)	9.3 (1.5)	8.9 (1.3)	9.4 (1.6)	0.14
LVEDV (mL)	127 (25.7)	132.5 (3.5)	125.4 (29.4)	0.56
LA dilation, n (%)	7 (9.6)	2 (8.3)	5 (10.2)	1.00
Biatrial dilation, n (%)	2 (2.8)	0 (0.0)	2 (4.3)	0.55
LAVI (mL/m2)	28 (8.5)	27.1 (5.7)	28.5 (9.6)	0.45
RV dysfunction, n (%)	2 (2.6)	1 (4.0)	1 (1.9)	0.55
RVSP (mm Hg)	27.5 (5.2)	28.8 (6.2)	26.6 (4.3)	0.18
E/e′^c^	7.7 (2.7)	8.3 (3.4)	7.4 (2.2)	0.25
LV GLS (%)	−20.5 (2.6)	−21.7 (2.1)	−19.8 (2.7)	0.005
Gene variants				
Val50Met, n (%)	25 (29.4)	11 (36.7)	14 (25.5)	0.28
Thr60Ala, n (%)	24 (28.2)	6 (20.0)	18 (32.7)	0.21
Val142Ile, n (%)	8 (9.4)	3 (10.0)	5 (9.1)	1.00
Ile107Val, n (%)	6 (7.1)	4 (13.3)	2 (3.6)	0.18
Other, n (%)	22 (25.9)	6 (20.0)	16 (29.1)	0.36

AI = artificial intelligence; AVB = atrioventricular block; CKD = chronic kidney disease; ECG = electrocardiography; GLS = left ventricular global longitudinal strain; Hb = hemoglobin; LV = left ventricle; LVEDV = left ventricle end-diastolic volume; LVEF = left ventricle ejection fraction; LA = left atrium; LAVI = left atrium volume index; NT-proBNP = N-terminal pro–B-type natriuretic peptide; PLT = platelets; PYP = pyrophosphates; RV = right ventricle; RVSP = right ventricle systolic pressure; WBC =white blood cell.

Data represent mean (SD) or number (percentage) unless otherwise specified.

a The mean difference of continuous variables between 2 groups with or without follow-up studies was tested using a two-sided t test. The association between categorical variables and the availability of follow-up was evaluated using χ2 or Fisher exact test as appropriate.

b Data represent median (1st quantile, 3rd quantile).

c We reported the mean between septal and lateral E/e′ when both were available. If lateral E/e′ was not available, the only septal E/e′ was reported.

ECG abnormalities included atrioventricular block (n = 3), atrial fibrillation (n = 3), low voltage (n = 1), and pseudoinfarct pattern (n = 3). The median AI-enabled ECG probability of CA was 6.3% (Q1-Q3 1.9-19.3), and 5 patients had an AI-ECG probability above the threshold indicating the presence of CA. However, none of these patients with a probability above the threshold had echocardiographic abnormalities at baseline.

At baseline echocardiographic assessment, mean septal and posterior wall thickness were 9.3 ± 1.5 and 9.1 ± 1.4 mm, respectively, mean left ventricular (LV) end-diastolic volume was 127 ± 25.7 mL, and LV ejection fraction (LVEF) was 62.6% ± 7.4%. A sensitive indicator of systolic dysfunction, LV global longitudinal strain, was slightly reduced (range −15.0% to −17.0%) in 11.5% of patients (n = 7/61), but overall mean values were normal at −20.5% ± 2.6%. Left atrial dilation was reported in 9.6% of subjects with a mean left atrium volume index that was normal at 28 ± 8.5 mL/m^2^. Mean LV filling pressures were not significantly elevated (7.7 ± 2.7). None of the patients had echocardiographic features suggestive of ATTR-CM. PYP scintigraphy at baseline was also normal in all patients assessed.

The *TTR* P/LP variants observed in our population are listed in Supplemental Table 1, with Val50Met being the most prevalent (29.4%), followed by Thr60Ala (28.2%), Val142Ile (9.4%), and Ile107Val (7.1%).

### Clinical progression in ATTR G+ carriers

Among the 85 G+ carriers, follow-up, clinical, and imaging studies were available in 55 patients over a median time of 6.8 years (Q1-Q3 4.1-9.7). Almost all the baseline characteristics are not statistically significantly different between the population with or without follow-up cardiac imaging (Table 1). Within the group with follow-up available, 53 patients had echocardiography and 20 patients had a PYP imaging (Supplemental Table 2). Clinical and echocardiographic progression in this cohort is described in Table 2. Over the follow-up period, the risk of developing peripheral neuropathy was 25.5% (95% CI: 8.9, 42.1%) as compared to baseline (*P* = 0.004). Moreover, there were an additional 4 patients who developed spinal stenosis, 7 bilateral carpal tunnel syndrome, 5 atrial fibrillation, and 6 syncope. Among the patients (n = 24) who were diagnosed with peripheral neuropathy, 15 underwent skin/nerve biopsy and 2 patients stained positive for amyloid deposition. None of the patients were diagnosed with heart failure during the follow-up period.

**Table 2:** Clinical and Echocardiographic Characteristics in G + ATTRv Family Members With Follow-Up Data Available (N = 55)

	Baseline	Follow-Up	Difference	*P* Value
Comorbidities				
Spinal stenosis, n (%)	2 (3.6)	6 (10.9)	7.3 (−2.3, 16.9)	0.14
Peripheral neuropathy, n (%)	10 (18.2)	24 (43.6)	25.5 (8.9, 42.1)	0.004
CKD, n (%)	1 (1.8)	6 (10.9)	9.1 (0.1, 18.1)	0.051
Bilateral carpal tunnel syndrome, n (%)	9 (16.4)	16 (29.1)	12.7 (−2.8, 28.2)	0.11
Atrial fibrilation, n (%)	3 (5.5)	8 (14.5)	9.1 (−2, 20.2)	0.11
Pacemaker, n (%)	1 (1.8)	1 (1.8)	-	-
Syncope, n (%)	2 (3.6)	8 (14.5)	10.9 (0.4, 21.5)	0.047
Echocardiography				
LVEF	63.3 (4.3)	61.2 (3.6)	−2 (−3.6, −0.4)	0.014
Septal wall thickness (mm)	9.4 (1.6)	10.2 (2.4)	0.7 (0, 1.3)	0.037
Abnormal septal wall thickness, n (%)^a^	0 (0)	4 (8.7)	8.7 (0.6, 16.8)	0.035
Posterior wall thickness (mm)	9.3 (1.5)	9.2 (2.2)	0 (−0.5, 0.6)	0.85
Abnormal posterior wall thickness, n (%)^a^	0 (0)	2 (4.5)	4.5 (−1.6, 10.7)	0.12
Left atrium volume index (mL/m^2^)	28.5 (9.6)	29.2 (6.8)	1.1 (−2.3, 4.5)	0.51
Left atrium dilation, n (%)	5 (10.2)	14 (29.8)	19.6 (4, 35.2)	0.016
Biatrial dilation n, (%)	2 (4.3)	4 (8.9)	4.6 (−5.5, 14.8)	0.36
E/e′^b^	7.4 (2.2)	7.2 (2.4)	0.3 (−0.6, 1.1)	0.55
LV GLS (%)	−19.8 (2.7)	−20.4 (3)	−0.6 (−1.5, 0.3)	0.20

Values are mean (SD) or number (percentage) unless otherwise specified. The proportional difference (95% CI) for binary variables or the mean difference (95% CI) for continuous variables is reported in the column named “Difference”. For binary variables, the proportional difference between follow-up and baseline levels was tested to see if it is different from 0. For continuous variables, a two-sided paired t test was used to test if the variable’s mean is different between baseline and follow up time. The *P* value obtained from statistical tests is reported. Bonferroni correction was used to account for multiple testing. Therefore, the significance level is 0.05, and the adjusted significance level is 0.05/16 = 0.00313.

Abbreviations as in Table 1.

a It was considered abnormal if >13 mm.

b We reported the mean between septal and lateral E/e′ when both were available. If lateral E/e′ was not available, the only septal E/e′ was reported.

### Cardiac disease progression in ATTR G+ carriers

A mild reduction in LVEF (63 ± 4-61 ± 4; *P* = 0.014) with thickening of the septal wall (9.4 ± 1.6-10.2 ± 2.4; *P* = 0.037) was observed, likely driven by the 4 patients who had abnormally thickened septal walls detected at the follow-up. In addition, there were 9 patients who developed left atrial dilation during the follow-up. We did not observe a progression in mean LV filling pressures or abnormal global longitudinal strain (Table 2).

There were 6 patients who had at least 2 echocardiographic changes suggestive of ATTR-CM. Specifically, 3 patients exhibited a significantly thickened septal wall, with LV global longitudinal strain values below −18.0%. One patient developed septal thickening (from 12 to 16 mm) accompanied by biatrial dilation. The other 2 patients developed biatrial dilation with the presence of elevated LV filling pressures (E/e′ rising from 7 to 15 in 1 case and to 10 in the other).

Five patients developed a positive PYP scan during the follow-up. They were diagnosed at the ages of 69, 67, 62, 43, and 41 years, and their genotypes were Val50Met, Thr60Ala, Thr60Ala, Gly67Glu, and Glu74Ser, respectively. Four of these 5 patients had echocardiography available at follow-up. Interestingly, none of these patients other than one had developed echocardiographic features of CA.

Among the 5 patients who had a positive AI-ECG for CA at baseline, 4 had cardiac imaging available at follow-up. Specifically, among these, 3 patients developed abnormal septal wall thickness, whereas the fourth had an increase from 9 to 12 mm. Among the remaining 49 patients with echocardiography at follow-up and a negative AI-ECG for CA at baseline, only one of them developed abnormal LV wall thickness.

## Discussion

This novel study, the largest of its kind to describe ATTRv G+ carriers at initial assessment with a comprehensive longitudinal cardiac imaging follow-up, sheds light on the complex variable dynamics of cardiac disease penetrance and expressivity in ATTRv CA. These findings could help counsel ATTRv G+ individuals and may guide informed decision-making regarding the timing of initiating a medical therapy. This study also provides insights into the evolution of cardiac morphology and function as assessed by echocardiography in this genetically diverse group of *TTR* carriers (Central Illustration).

**Central illustration:**
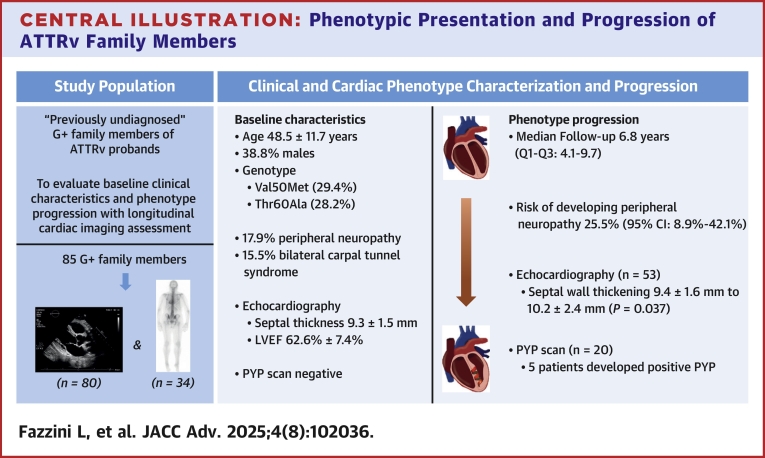
**Phenotypic Presentation and Progression of ATTRv Family Members** We explored the study population’s baseline features and longitudinal findings in asymptomatic ATTRv family members. At baseline, individuals had normal echocardiographic and pyrophosphate scan profiles. Over the follow-up, we described clinical and echocardiographic progression. In patients with follow-up bone scintigraphy, 5 developed a positive scan. This study highlights the features of progression over time and supports the importance of structured surveillance in *TTR* variant carriers. ATTRv = hereditary transthyretin amyloidosis; *TTR* = transthyretin.

Among genetic CMs, the time from being diagnosed with a P/LP to developing the disease is highly variable and often undefined.^20^, ^21^, ^22^, ^23^ A large dataset demonstrated that the frequency of a P/LP *TTR* variant is not so rare (approximately 1 in 1000 individuals).^24^ The development of ATTRv peripheral neuropathy varies based on geographical regions, genotypes, and among families.^25^, ^26^, ^27^ Similarly, ATTRv peripheral neuropathy is also characterized by variable expressivity of cardiac disease.^4^ A relationship between age at diagnosis and disease penetrance has been described, although the underlying age-dependent mechanisms are unknown.^28^ Male sex has also been associated with an increased risk for the development of peripheral neuropathy in ATTRv 50 M carriers.^25^ Although the natural history of ATTRv carriers has been previously described, this study was based on the occurrence of symptoms alone as assessed by an observational survey and had a median follow-up of only 2 years.^29^ The outcomes of “asymptomatic” patients (defined as the absence of heart failure symptoms) but with baseline echocardiographic evidence of ATTR-CM (unlike our cohort) have also been previously described.^30^ In addition, ATTRv carriers enrolled in the UK Biobank were found to be at a higher risk of heart failure.^24^ However, initial presentation and subsequent cardiac disease progression and expressivity based on longitudinal cardiac imaging in G+ family members before this study were largely unknown.^31^ In the Atherosclerosis Risk in Communities study, V122I *TTR* variant Black carriers (n = 124) were found to be at an increased risk of incident heart failure as compared to noncarriers over a 21.5 years of follow-up.^32^ Recently, V122I carriers who were predominately Black were described to have a higher risk of all-cause mortality and heart failure hospitalizations, particularly driven by heart failure with reduced ejection fraction, compared to noncarrier controls.^33^ However, in both these studies, whether CA was present at baseline and whether heart failure occurred due to CA or other causes such as coronary artery disease or hypertension were unknown. Furthermore, White subjects were not studied, given that the V122I variant is predominantly present in Blacks. Our study addresses these limitations by describing the disease-specific natural history of ATTRv by cardiac imaging in G+ patients who mostly had variants other than V122I.

It is relevant to highlight that 18% of our overall cohort of family members had a history of clinically diagnosed peripheral neuropathy at baseline and an additional 26% of patients developed neuropathy subsequently. The high prevalence and subsequent development of peripheral neuropathy rather than symptomatic CM underscores the potential neurotropic nature of ATTRv for the genotypes reported.

Progressive increase in LV wall thickness, a classic echocardiographic feature of CA, and a reduction in LVEF were observed, but the latter variable remained within normal limits in these patients. Sensitive echocardiographic deformation parameters such as myocardial strain have been proposed to detect early involvement of the heart in amyloidosis. However, these values also remained within the normal range in our population. Myocardial deformation echocardiographic imaging has primarily been validated in cohorts with some grade of increased myocardial wall thickness, and whether this echocardiographic parameter could be useful in the early identification of ATTR-CM in a G + population prior to this study was unknown.^34^

It is unknown whether and when to initiate newer available therapy for amyloidosis in asymptomatic newly diagnosed ATTRv G+ patients. Identifying such asymptomatic G+ individuals with a high probability of developing subsequent symptomatic disease represents an unmet need in clinical practice. In this context, we applied a previously validated AI-enabled ECG algorithm, which has demonstrated high diagnostic performance for CA in prior studies with an area under the receiver operating characteristic curve of 0.91. AI-ECG could be a valuable, low-cost, and noninvasive screening modality that may be capable of identifying early or subclinical cardiac involvement, as compared to conventional ECG interpretation. Its use in this asymptomatic genetically at-risk population was based on its standard availability in our clinical practice as part of the electronic health record and evaluating its potential role in enabling risk stratification. Patients who are identified as high risk may warrant more frequent follow-up with multimodality imaging. Important limitations must be acknowledged. The algorithm was primarily validated in populations with clinically overt CA; thus, although AI-ECG holds promise in informing early diagnostic and preventive strategies, its clinical utility in this subset of patients remains exploratory and requires prospective validation.

### Patient perspective

Returning genetic testing results can be emotionally and logistically challenging for both family members and health care providers given the uncertainty in outcomes.^35^ Genetic testing results might have a profound impact on individuals and families, highlighting the need for such studies and high-touch methods such as one-on-one genetic counseling. Hopefully, our study with others will help inform not only the clinicians who encounter these patients but also the patients themselves.

### Study Limitations

A limitation of our study is the relatively small sample size, which is an inherent limitation of rare diseases, although it is the largest study of ATTRv carriers with longitudinal cardiac imaging follow-up that provides information on the development and progression of cardiac disease in previously undiagnosed ATTRv carriers. Although a longer period of follow-up may have resulted in a higher incidence of cardiac disease, this study with a median 6.8-year follow-up period provides a reasonable estimate of disease impact for clinical purposes. Furthermore, given the retrospective nature of the study, only a subset of patients underwent PYP scintigraphy, which is known to be more sensitive than echocardiography for detecting ATTR-CM. However. whether therapy should be initiated in asymptomatic ATTRv patients with a positive PYP scan but without evidence of structural heart disease by echocardiogram is unknown. In addition, as no standardized imaging follow-up protocol was in place, the timing and modality of follow-up cardiac imaging was based primarily on clinician discretion. Actual imaging follow-up varied across patients, which we speculate may have been due to patient-specific clinical judgment, insurance coverage limitations, logistical constraints, and patient preference. Cardiac disease penetrance and progression may be affected by a specific *TTR* genotype; however, the heterogeneity of genotype in our study population limits our ability to infer conclusions based on genotype. Finally, our study is subject to inherent limitations of retrospective designs, including potential selection bias related to a single tertiary care center setting involving a predominantly White group of patients, and information bias due to missing data and variability in clinical assessment and follow-up. These factors may limit the generalizability of our findings.

## Conclusions

Previously undiagnosed ATTRv G+ family members have a higher prevalence of neurological than cardiac disease at the time of genetic testing. During a median follow-up of 6.8 years, symptomatic cardiac involvement is rare, and these patients are more likely to develop neuropathy than heart failure. These findings could assist in informing and counseling G+ family members about ATTRv cardiac progression and provide clinicians with a cardiac disease trajectory, which may be helpful in the timing of initiating an appropriate therapy for ATTRv-CM. There is a need to develop disease-predictive tools for asymptomatic ATTR G+ patients by establishing larger longitudinal registries of these patients.

Perspectives **COMPETENCY IN MEDICAL KNOWLEDGE:** Given the increasing adoption of genetic screening, newer imaging, and therapeutic options, it is important to understand the onset and development of ATTRv-CM in previously undiagnosed G+ individuals. Genetic screening of the ATTRv proband’s family members enables a diagnosis of previously unrecognized peripheral neuropathy due to amyloid. Longitudinal clinical follow-up findings described in this study could help counsel these patients while offering clinicians valuable insights into the trajectory of symptomatic cardiac disease if any.

**TRANSLATIONAL OUTLOOK:** The disease progression described in this study requires validation in a large-scale registry of such patients to develop disease prediction tools and to inform the design of clinical trials and potential therapeutic interventions for such patients.

## Funding support and author disclosures

Dr Fazzini received a grant funded by the 10.13039/501100021857Italian Society of Cardiology and Bruno Farmaceutici. All other authors have reported that they have no relationships relevant to the contents of this paper to disclose.
