# Supplementary material for: Phenotypic Presentation and Longitudinal Characterization of Hereditary ATTRv Amyloidosis in Previously Undiagnosed Family Members
Source: JACC Adv. 2025 Jul 24;4(8):102036. doi: 10.1016/j.jacadv.2025.102036 (PMC12311500; doi:10.1016/j.jacadv.2025.102036)

Supplemental Table 1. All transthyretin variants in G+ ATTRv family members.

TTR variants	Patients
Val30Met	25
Thr60Ala	24
Val122Ile	8
Ser77Tyr	7
Ile107Val	6
Glu74Ser	3
Leu58His	3
Pro24Ser	3
Gly67Glu	1
Ile84Ser	1
Phe64Leu	1
Ser50Arg	1
lle88Leu	1
Tyr134Cys	1

Supplemental Table 2. Modality of imaging at baseline and follow-up.

**Baseline (n=55)**	**Follow-up (n=55)**
Echocardiography (n=53)	Echocardiography (n=48)
	PYP scintigraphy (n=31)
	Cardiac Magnetic Resonance (n=3)
PYP scintigraphy (n=20)	Echocardiography (n=16)
	PYP scintigraphy (n=14)
	Cardiac Magnetic Resonance (n=0)
Cardiac Magnetic Resonance (n=4)	Echocardiography (n=5)
	PYP scintigraphy (n=0)
	Cardiac Magnetic Resonance (n=1)

Supplemental Figure 1. Patient's selection flow diagram.

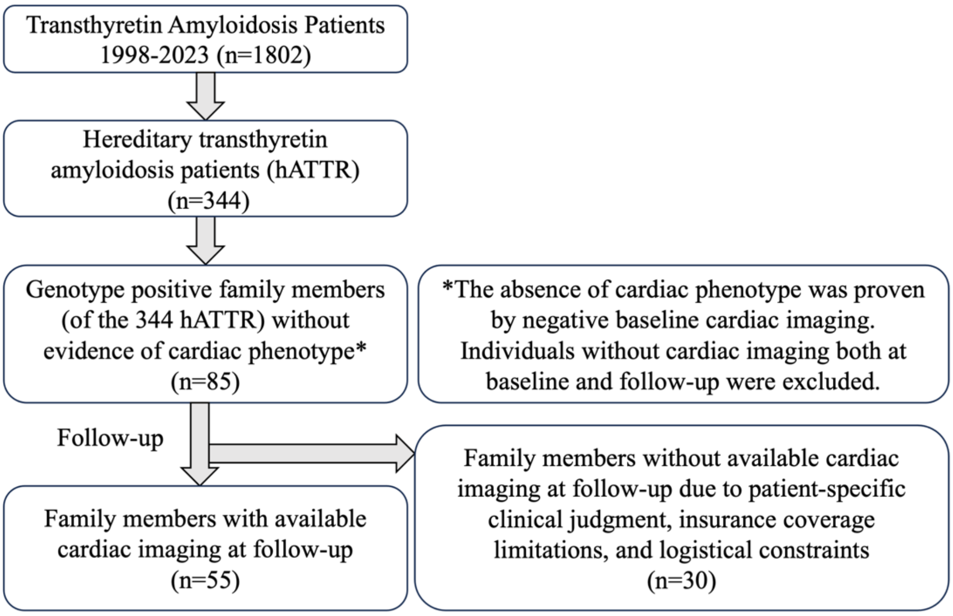

Supplement: Supplemental_Material [file mmc1.pdf]
